# Transcriptional analysis of *ftsZ* within the *dcw* cluster in *Bacillus mycoides*

**DOI:** 10.1186/1471-2180-13-27

**Published:** 2013-02-06

**Authors:** Tiziana Santini, Luana Turchi, Giulia Ceccarelli, Carmen Di Franco, Elena Beccari

**Affiliations:** 1 Dipartimento di Biologia e Biotecnologie “C. Darwin”, Rome, 00185, Italy; 2 Istituto di Biologia e Patologia Molecolari IBPM-CNR, Università Sapienza, Roma, P.le A. Moro 5, Rome, 00185, Italy

**Keywords:** *B. mycoides*, *dcw* cluster, *ftsZ*, Initiation of transcription

## Abstract

**Background:**

In *Bacillus mycoides*, as well as in other members of the *B. cereus* group, the tubulin-like protein of the division septum FtsZ is encoded by the distal gene of the cluster *d*ivision and *c*ell *w*all *(dcw)*. Along the cluster the genes coding for structural proteins of the division apparatus are intermingled with those coding for enzymes of peptidoglycan biosynthesis, raising the possibility that genes with this different function might be coexpressed. Transcription of *ftsZ* in two model bacteria had been reported to differ: in *B. subtilis,* the *ftsZ* gene was found transcribed as a bigenic mRNA in the AZ operon; in *E. coli,* the transcripts of *ftsZ* were monogenic, expressed by specific promoters. Here we analyzed the size and the initiation sites of RNAs transcribed from *ftsZ* and from other cluster genes in two *B. mycoides* strains, DX and SIN, characterized by colonies of different chirality and density, to explore the correlation of the different morphotypes with transcription of the *dcw* genes.

**Results:**

In both strains, during vegetative growth, the *ftsZ-*specific RNAs were composed mainly of *ftsZ, ftsA-ftsZ* and *ftsQ-ftsA-ftsZ* transcripts. A low number of RNA molecules included the sequences of the upstream *murG* and *murB* genes, which are involved in peptidoglycan synthesis. No cotranscription was detected between *ftsZ* and the downstream genes of the *SpoIIG* cluster. The monogenic *ftsZ* RNA was found in both strains, with the main initiation site located inside the *ftsA* coding sequence. To confirm the promoter property of the site, a *B. mycoides* construct carrying the *ftsA* region in front of the shortened *ftsZ* gene was inserted into the *AmyE* locus of *B. subtilis* 168. The promoter site in the *ftsA* region was recognized in the heterologous cellular context and expressed as in *B. mycoides*.

**Conclusions:**

The DX and SIN strains of *B. mycoides* display very similar RNA transcription specificity. The *ftsZ* messenger RNA can be found either as an independent transcript or expressed together with *ftsA* and *ftsQ* and, in low amounts, with genes that are specific to peptidoglycan biosynthesis.

## Background

*Bacillus mycoides*, a Gram positive soil rod bacillus of the *B. cereus* species-group [1], is characterized by hyphal colonies with cells connected at the poles in long filaments. These filaments converge into bundles that mainly curve clock- or counter-clockwise in two kinds of bacilli, both of which were attributed to *B. mycoides*[2].

We have previously isolated [3] examples of the two types from the environment and followed the process of colony formation on agar of two strains, i.e. DX with the right-curving colony branches and SIN with the left-curving colony branches. The initial cell filaments formed after seeding the cells on agar already showed the strain-specific turn direction and the colonies in the advanced growth stage appeared with a different overall density. The differences prompted a genetic characterization of the strains beyond the identical metabolic properties detected by monitoring 50 enzymatic reactions using the API50CH test. Genomic similarity of DX and SIN was thus checked by examining the region of the *dcw* (*d*ivision *c*ell *w*all) cluster, composed of a group of fundamental genes coding for several proteins of the division apparatus and for enzymes of peptidoglycan biosynthesis [3]. The distribution in the cells of the sites of new peptidoglycan synthesis, which was also analyzed in these strains, was found to be very similar [4]. A very limited number of DX and SIN nucleotides differs along the *dcw* region. This points to a close evolutionary relationship between the two strains as well as between the members of the *B. cereus* group. Comparative genome analysis of a large number of bacilli attributed to the group recently led to the proposal that they should be classified as a single species [1].

Here we extended sequencing to additional genes of the cluster and, in order to better characterize these different strains, we examined the RNAs expressed in vegetative cells. In particular, we focused on the specific transcripts of the genes coding for two proteins, FtsZ and FtsA, which are the building blocks of the Z ring assembly for septum formation during cell division. Among the various bacilli, the expression of these two genes was examined only in *B. subtilis*[5,6]. Both papers reported that *ftsA* and *ftsZ* form an operon, transcribed as a bigenic *ftsA-ftsZ* RNA. In the Northern blot shown by Gholamhoseinian et al. [5], the *ftsZ* probe binds to a band with the length of a single-gene transcript, but it was not investigated further because it was considered as a degradation product. We found instead that in both *B. mycoides* strains, in addition to polycistronic transcripts, *ftsZ* is transcribed as the single-gene RNA, independently of *ftsA*.

## Results and discussion

### Northern blot analysis of transcripts

**Figure 1:**
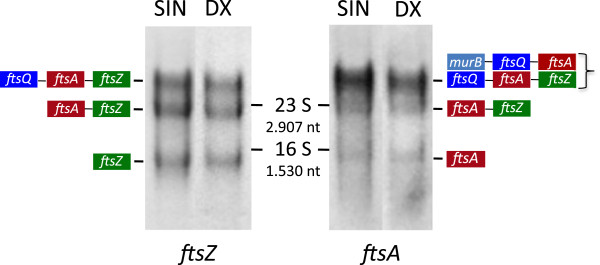
**Northern blot analysis of RNA from exponentially growing *B. mycoides *SIN and DX.** SIN and DX total RNA was electrophoresed in formaldehyde-agarose and blotted. The same filter was hybridized first to *ftsZ* and, after stripping, to *ftsA* DNA probes. The position of ribosomal 23S (2907 bases) and 16S (1530 bases) RNA on the filter is indicated. *FtsZ* and *ftsA* RNAs in the band below 16S rRNA are monogenic transcripts. The band below the position of the 23 S rRNA contains the *ftsA-ftsZ* bigenic transcripts. The transcripts of the genes *ftsQ-ftsA-ftsZ* are within the uppermost bands together with the transcripts *murB-ftsQ-ftsA,* detected only by the *ftsA* probe.

In *B. mycoides*, *ftsA* and *ftsZ* occupy the 3’ end of the *dcw* cluster, separated by 39 bp of non-coding DNA. Transcripts of these two genes were sized in Northern blots of SIN and DX vegetative RNA (Figure 1).

The *ftsZ* DNA probe detected three main RNA components in SIN and DX: the shortest one, found just below the position of the 16S *B. mycoides* ribosomal RNA (1530 nucleotides), was the size of the monogenic transcript, since the *ftsZ* coding region spans 1155 nucleotides from the starting ATG to the termination triplet; the second hybridization band, below the 23S ribosomal RNA (2907 nucleotides), harbored transcripts with the length of *ftsA* plus *ftsZ*; the third band, in the upper part of the gel, carried RNAs corresponding to the size of three genes, thus *ftsQ-ftsA-ftsZ* since *ftsZ* is not cotranscribed with downstream genes.

The *ftsA* probe, hybridized to the same filters, revealed three *ftsA*-specific RNA bands. The fastest one migrated slightly less than the monogenic *ftsZ* RNA band, which is in keeping with the 144 bp longer coding sequence of the *ftsA* gene; the second *ftsA*-specific band colocalized with the *ftsZ* bicistronic transcripts; the third band in the uppermost position was broader and more intense than the other two bands, indicating that *ftsA* was particularly abundant in long transcripts, mostly *ftsQ-ftsA-ftsZ* RNA. The intensity of the uppermost band is higher when probed with *ftsA* than when probed with *ftsZ,* indicating that a fraction of the transcripts does not contain *ftsZ* but carries the RNA of the *murB* gene, located upstream of *ftsQ* (Figure 1, schematics).

These results show that the bulk of the *ftsA* and *ftsZ-*specific RNAs were in molecules spanning one, two and three gene units, though the low level of detection and molecular weight definition of the Northern blots required further analysis.

### Primer extension analysis of *ftsZ*, *ftsA* and *ftsQ* RNA

**Figure 2:**
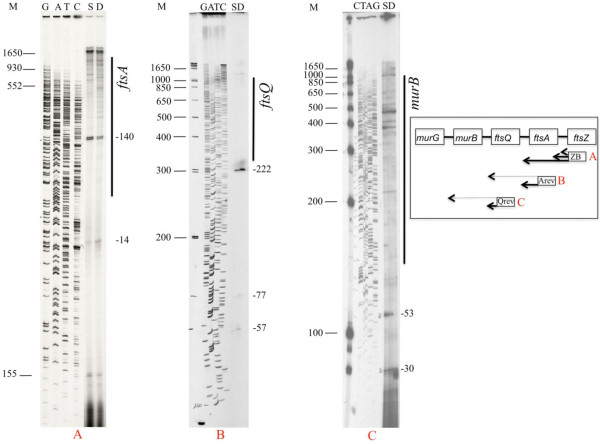
**Determination of *ftsZ, ftsA *and *ftsQ *RNA 5’ ends by primer extension (PE) in *B. mycoides *SIN (S) and DX (D).** 5’ ^32^P-labeled primers were hybridized to total RNA, extended by reverse transcriptase and the cDNAs separated by 6% urea-PAGE electrophoresis. The numbers on the right side of the autoradiograms indicate the position of the cDNA 3’ ends relative to the ORF first nucleotide (+1). The thick lateral bar indicates the approximate position in the gel of the next upstream gene. **A**) PE from primer ZB annealing to *ftsZ* RNA at +103; **B**) PE from primer Arev annealing to *ftsA* RNA at +80; **C**) PE from primer Qrev annealing to ftsQ RNA at +52 (Table 1). Here two different SIN cDNA preparations were loaded on the gel. A schematic view of the major cDNA products is shown in the inset. M = MW marker ^32^P-labeled DNAs. GATC = ^35^S-dATP labeled M13mp18 ladder.

In order to map the initiation sites of the observed RNAs, the vegetative SIN and DX RNAs were analyzed by Primer Extension (PE) (Figure 2). *FtsZ* transcripts were hybridized to primer ZB (Table 1), annealing to RNA at nucleotide position +103 relative to the A of the first ATG codon of the *ftsZ* open reading frame (+1). Two cDNA bands, elongated by reverse transcriptase (RT) starting from this primer, stopped at positions −14 and −140 (Figure 2A and Additional file 1). The −140 cDNA, which mapped inside the coding sequence of the preceding gene *ftsA,* was more abundant than the one at −14. The fact that the −14 position lies in the spacer region between *ftsA* and *ftsZ,* at the upper end of the ribosome binding site (RBS), suggests that this RNA may originate from a longer RNA, such as the one at −140, protected from degradation by ribosomes bound to the RBS.

**Table 1:** Primers used in this study

***Primer name***	***Primer sequence***	***gene***	**(a)**
**Northern probes**			
Zfor	AAAGTWATCGGTGTCGGCGGWGGC	*ftsZ*	+43
Zrev	CAGAAATACCTTGAACCCCTTGGCG	*ftsZ*	+595
Ain	GAACAGCAATGAAATATATGTTG	*ftsA*	+3
N2R	ACCGTCTACAATGAACTGTC	*ftsA*	+411
**Primer Extension**			
prex	GCCCAAACCGCACTCGCAC	*ftsW*	+95
Wrev	AATCCATTCTCTGTACCAATG	*murG*	+125
Rip2	GTTGCTTAGYAGCCAGTTTC	*murG*	+1030
Qrev	TCTTTARCTTTGGTACACGATC	*ftsQ*	+52
Arev	TCATTAACCATTTCACCAATGATG	*ftsA*	+80
N2R	ACCGTCTACAATGAACTGTC	*ftsA*	+411
ZB	CACCGTGTTCAATCATACGG	*ftsZ*	+103
ZD	ACAACCAAACAACGTCGGCG	*spoIIGA*	+74
ZDbis	CCTAACACAAGCCTCCATC	*spoIIGA*	+158
BigD	CCCAAATGCTGTATACACAATAAGTAACGAG	*spoIIGA*	+273
**RT-PCR**			
Zfin	CTTTTATCGTCTACGACGGTTAC	*ftsZ*	+1158
Zin	CATGTTAGAGTTTGATACTACTC	*ftsZ*	−1
Ain	GAACAGCAATGAAATATATGTTG	*ftsA*	+3
Afin	CCCATAAATAACGGAATGCACG	*ftsA*	+1297
Qin	CGTACATGAARAAYAGTAARG	*ftsQ*	−5
Mbin	GAGATTGTCTATGGAACAATTAG	*murB*	−10
MGin	ACAGCTGAAACNCTTATTCGTG	*murG*	+964
Fw	CATCAGCACCGTATCGRATG	*ftsW*	+601
**Mini-ftsZ**			(b)
Hind5	GAC**AAGCTT**ATATTGGTGTTCGTGAG	*ftsA*	+1056
Eco5	GGC**GAATTC**GCTAATTGATCTTGAG	*ftsZ*	+39
Eco3	CAC**GAATTC**AAAACAACGTGAAGTTAAG	*ftsZ*	+1035
Bam3	GGC**GGATCC**AAAAAGGAGCATGAAAGCTC	*spacer*	+28
Amy5	GCCGCGATTTCCAATGAGG	*pJPR1*	+245

(a) Position of the primer 5’ nucleotide on the corresponding gene numbering beginning from the first codon of the gene (+1).

(b) Position on the gene of the first complementary primer base after the added restriction site evidenced bold.

cDNA bands were also detected in a gel position close to the 1650 bp MW marker, thus mapping within the spacer region between *ftsA* and the upstream gene *ftsQ.* Additional bands were visible in the upper part of the sequencing gels, where compression does not allow size definition. These data indicate that *ftsZ* is transcribed as a monogenic RNA and a bigenic *ftsA-ftsZ* RNA, thereby confirming the Northern blot data.

Initiation sites of *ftsA*-specific RNAs were analyzed by PE from primer Arev (+ 80 in *ftsA*, Table 1). Three minor cDNAs mapped at −9, -57 and −77 and a major one at −222 from the first nucleotide of the *ftsA* ORF, all of them within the 400 bp spacer region between *ftsQ* and *ftsA* (Figure 2B and Additional file 1). The major −222 RNA transcript resembles the vegetative P3 transcript of *B. subtilis* initiating at −285 from the *ftsA* ORF [6]. The −222 start site is preceded by the same modules for *sigmaA* recognition as the *B. subtilis* promoter, mapped within the *sbp* gene that separates *ftsQ* from *ftsA* in *B. subtilis*. In *B. mycoides,* there is no open reading frame in the *Q-A* spacer region, but only similarity to *B. subtilis sbp* in short dispersed sequences*.*

Figure 2C shows the *ftsQ*-specific cDNAs extended from primer Qrev (+52, Table 1). cDNA elongation stopped at nucleotide −30 both in SIN and DX. The two strains differed in this location insofar as a cDNA band was present at −27/28 in DX alone and one at −53 in SIN alone. In this region, one base difference between the two strains changes the stability of a stem composed of two inverted repeats of 11 nucleotides. Several cDNA ends, which were either strain-specific or common to both strains, were visible within the upstream *murB* gene sequences. The RNA initiation sites located upstream of *murB* indicate the cotranscription of *ftsQ* with *murB* and probably with *murG,* though gel compression prevents a precise length determination of the cDNAs.

### RT-PCR analysis of *dcw* transcripts

**Figure 3:**
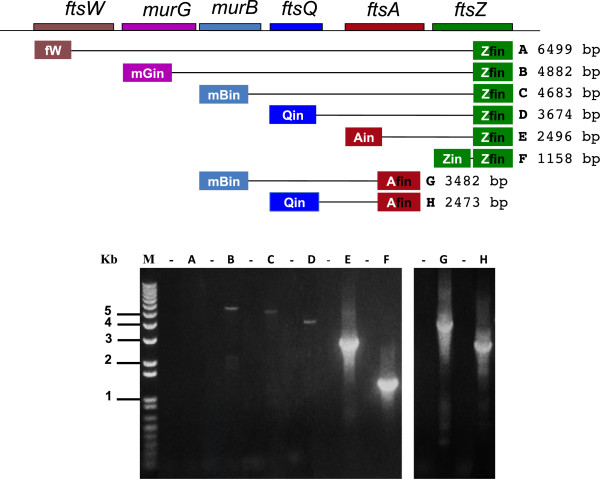
**RT-PCR analysis of RNA transcripts from the *dcw *genes in *B. mycoides*.** Purified vegetative RNA of *B. mycoides* DX was reverse transcribed from primers complementary to the 3’ end of *ftsZ* (Zfin) and to the 3’ end of *ftsA* (Afin). The control cDNAs (lanes -) were without RT in the reaction. cDNAs were PCR amplified using Zfin (**A-F**) and Afin (**G-H**) as downstream primers. Upstream primers were specific for each gene (Table 1). Multigene *ftsZ* RNAs included *murG* and *murB*, though not *ftsW* transcripts.

The high MW transcripts were instead highlighted by RT-PCR analysis (Figure 3). Using *B. mycoides* RNAs controlled for the absence of DNA, cDNA was synthesized from the Zfin primer which is complementary to the 3’end of *ftsZ.* PCR amplifications of the cDNA were then produced using this downstream primer and descending primers from each of the sequenced *B. mycoides dcw* genes (Table 1). The longest amplification product (lane B of the agarose gel) indicated the existence of RNA transcribed from 5 genes, *murG, murB, ftsQ, ftsA* and *ftsZ.* The PCR did not detect molecules including *ftsW*/*spoVE* sequences (lane A)*.*

The cDNA prepared using the primer Afin, complementary to the end of the ftsA gene, was also amplified using Afin as the downstream primer and upstream primers specific for *murB* and for *ftsQ* (Figure 3, lanes G, H). Although a simple PCR does not provide a precise quantification, the *murB-ftsQ-ftsA* RNA and the *ftsQ-ftsA* RNA are better represented than the RNA *ftsQ-ftsA-ftsZ*, which is in accordance with the Northern blot data.

The continuous coverage by RNA transcripts of the *dcw* cluster from *murG* to *ftsZ* has recently been reported in another member of the *B. cereus* group, the *B. anthracis Ames ancestor*, in the study of the whole genome transcriptome. The shotgun sequencing of cDNA (RNA-Seq) obtained from RNA transcribed under various growth conditions provided a map of transcription start sites and operon structure in the *B. anthracis* genome; in this study the *ftsZ* gene was found to be cooperonic with *ftsA*, *ftsQ*, *murB* and *murG*. [7].

### Heterologous expression of a *ftsZ* minigene

Monogenic transcripts of the *ftsZ* gene, guided by at least three promoters located within the *ftsA* coding region, have been described in *E. coli*[8]. In the Gram positive model bacillus, *B. subtilis*, the *ftsZ* RNA was only considered as a part of the bigenic transcript of the *AZ* operon, directed by the activity of three promoters of different growth phase specificity located upstream of *ftsA*[5,6]*.* In our assays, Northern blots and PE data indicated transcription of *ftsZ* as a single gene; thus we decided to search for a *bona fide* promoter upstream of the RNA start sites seen in the experiments.

When determined by the primer extension technique, the real initiation point of a messenger RNA can sometimes be uncertain owing to RNA processing or to premature termination of the reverse transcriptase at secondary structures of the RNA. Our hypothesis was that if a specific promoter drove transcription of the *ftsZ* monogenic RNA, this mechanism could work in a similar cellular context. We thus chose to insert the *B. mycoides* DNA region harboring the putative −140 and −14 *ftsZ* initiation sites at the chromosomal *amyE* locus of *B. subtilis*. The −140 site is within the 3’ coding region of *ftsA* and the −14 site in the spacer region between *ftsA* and *ftsZ* (Additional file 1*).*

**Figure 4:**
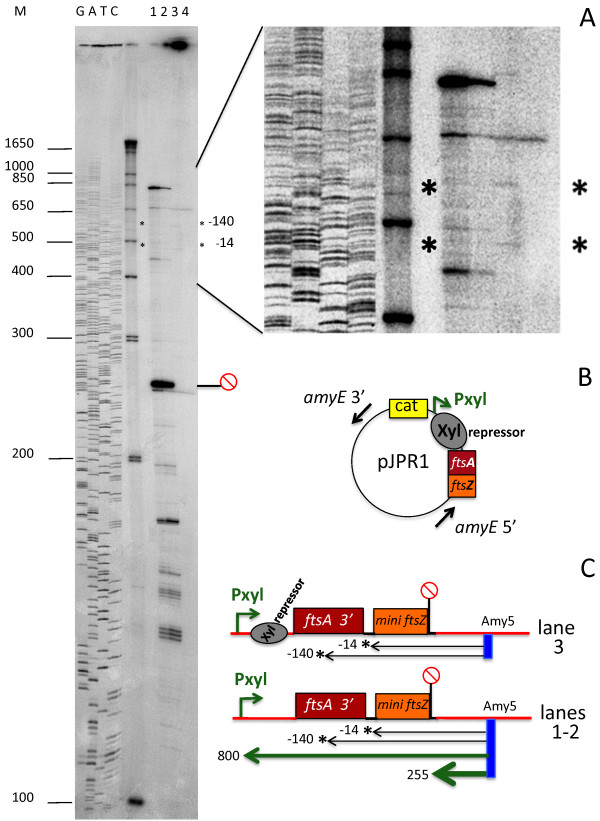
**Initiation of mini-*ftsZ *RNA transcripts in *B. subtilis*.** The *B. mycoides* mini-*ftsZ* DNA construct was cloned into pJPR1 and inserted at the *AmyE* site of *B. subtilis 168* (see methods). Transcripts of the construct were detected in total *B. subtilis* RNA by primer extension from the labeled primer Amy5 (Table 1) specific to the *amyE* 5’ region located 245 nt downstream of the inserted construct. **A**) Autoradiogram of PE. Lanes1 and 2: transcripts originating from the *Pxyl* promoter, induced by 5% xylose for 18 and 3 hours. Lane 3: the faint transcripts of the *ftsZ* minigene present in the non-induced *B. subtilis* recombinant strain are indicated by asterisks and map at −140 and −10 from the first nucleotide of the minigene *ftsZ* ORF as in *B. mycoides.* These bands are not present in the control *B. subtilis* strain (lane 4). **B**) schematic view of the construct in pJPR1. **C**) Schematic representation of the cDNAs indicated by asterisks in A. The red circle marks the position of the terminator structure 3’ to the *B. mycoides ftsZ* ORF. M = MW marker DNA. GATC = M13MP18 sequence ladder.

We created a shortened *B. mycoides* DX *ftsZ* gene, missing the central coding region, to make it easily distinguishable from the endogenous *B. subtilis* gene. The minigene was preceded by the 286 bp region containing the −140 and the −14 putative initiation sites and followed by 28 bp of the 3’ non-coding region after the *ftsZ* termination codon. The construct was inserted at the *B. subtilis* str.168 *amyE* locus after cloning into the pJPR1 integrative vector (*amyE:: Pxylcat*[9]). Plasmid pJPR1 carries the 5’ and 3’ regions of the *B. subtilis amyE* gene for integration of the recombinant sequences into the chromosome by a double cross-over. The sequences inserted into the plasmid cloning site and eventually integrated at the *amyE* site become controlled by the strong promoter *Pxy*l, which is induced by xylose but is normally blocked by a tight repressor (Figure 4B).

RNAs transcribed in the recombinant and wild type *B. subtilis* strains were analyzed by primer extension (Figure 4A), with the labeled primer Amy5 (Table 1) annealed to RNA of the 5’ *AmyE* region 245 nucleotides downstream of the minigene construct.

In addition to the aspecific bands present in both lanes, two faint but clear cDNA bands were detected in the recombinant (Figure 4A, lane 3) though not in the control *B. subtilis* (Figure 4A, lane 4). These bands are magnified in the lateral view. The longer cDNA (575 bp) maps at the nucleotide located at −140 bp from the starting ATG of the inserted mini-*ftsZ*, which is the same initiation site as that found for the RNA transcribed in *B. mycoides.* The second cDNA (465 bp) maps located in the short spacer region between *ftsA* and *ftsZ* containing the −14 site. The data show that the heterologous region is recognized by the *B. subtilis* transcription machinery as containing promoter elements and is hence transcribed as in the original context. As for the −14 RNA that starts at the RBS preceding the *ftsZ* ATG, it is still difficult to establish whether this shorter RNA is a maturation product of the longer RNA or an independent transcript.

When the *pxyl* promoter was induced by xylose for 18 hr (lane 1) and 3 hr (lane 2), strong cDNA bands were produced. The most intense band at position 255 is composed of a stop of the RT at the termination sequence located at the end of the *B. mycoides* mini-*ftsZ.* However, the RT also bypasses the terminator hairpin-loop structure and extends the cDNA up to the vector promoter site, forming the top band, which is about 800 bases in length. The lower bands are due to cDNA terminations in the vector sequences between the Amy5 primer and the minigene.

### Termination sequences

Transcription termination in *E. coli* is helped by specific proteins such as Rho [10], while Rho independent termination sites, in the form of RNA hairpins followed by a polyU stretch [11], are commonly found in Gram positive bacilli. The close parenthood of *B. mycoides* with the *B. cereus* group members prompted us to make use of the prediction program of Transcription Terminators, developed for Firmicutes, at the TransTerm-HP site [12]. The presumed termination sequences considered were those relative to *B. weihenstephanensis*[13]*,* the annotated genome with the highest similarity to the DX isolate. Only 34 nucleotide differences are present between DX and *B. weihenstephanensis* in the 10.731 bp *dcw* region we analyzed, while the number of nucleotide variations in the same DNA region is more than ten times greater comparing DX with other *B. cereus* group members. An additional element pointing to the close similarity of the two strains is the identity in length and in sequence of the very variable spacer region that separates the *dcw* cluster from the *SpoIIG* operon.

**Figure 5:**
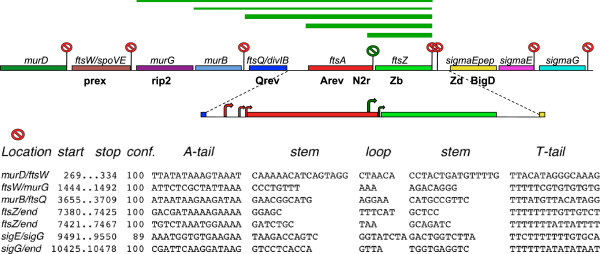
**Transcriptional terminators within the *B. mycoides dcw *and *spoIIG *gene clusters.** Red labels mark the position of the putative terminators. The DX termination sequences displayed are 100% identical to those predicted at the TransTerm-HP site for *B. weihenstephanensis* KBAB4 (Accession NC_010184, from coordinates 3780796 to 3790953)*.* The green label between *ftsA* and *ftsZ* indicates a hairpin structure not recognized there as a potential terminator. The three large green bars over the genes represent the main *ftsZ*-specific RNAs and the green thin bars the minor ones. The primers used to detect RNA 5’ ends by primer extension are indicated below the genes. The curved arrows in the enlarged region show the main *ftsA* and *ftsZ* RNA start sites.

The TransTerm-HP site had revealed several hairpin-loop structures in *B. weihenstephanensis,* with the specific characteristics of a terminator, in the spacer DNAs located between the gene coding regions. Figure 5 shows the location in the *dcw* and *SpoIIG* clusters of these putative terminators. The DNA sequences that form the structures are shown below the drawing. They are 100% identical in DX and in *B. weihenstephanensis*. Six out of seven are assigned a 100% confidence score by the algorithm of the program, and the seventh, between *sigmaE and sigmaG*, has an 89% score. The SIN termination structures are not identical, but maintain the characteristic of terminators with one or a few different nucleotides, the same level of diversity existing for instance between the terminators of *B. weihenstephanensis* and those of *B. anthracis Ames*.

The short 39 bp DNA region between *ftsA* and *ftsZ* can also be folded into a hairpin structure with a calculated stability of −7.8 ΔG, though it is not recognized as a potential terminator by the TransTerm-HP site and is tagged with a different color in the figure. Downstream of the *dcw* cluster, in the group composed of three genes, *SpoIIA-sigmaE processing peptidase*, *prosigmaE* and *sigmaG,* putative termination sequences are located between *prosigmaE* and *sigmaG* and after *sigma G,* at the end of the group.

The putative terminators are located at the boundary between genes of different specificity, which code either for enzymes of peptidoglycan biosynthesis or for structural proteins of the division septum, meaning that terminators are found between the *mur/fts* genes and not between the *mur/mur* or *fts/fts* genes. Two consecutive terminator hairpins close the *dcw* cluster immediately after the *ftsZ* gene.

In *B. anthracis,* another member of the *B. cereus* group, the genome-wide coverage of DNA by RNA transcripts has been analyzed at the single nucleotide level [7]. The high-throughput sequencing of total RNA (RNA-Seq), in various growth conditions, provided a map of transcript start sites and operon structure throughout the genome. Discontinuity of RNA transcripts in *B. anthracis,* along the *dcw* region shown in Figure 5 was observed in tracts corresponding to those harboring putative termination sites, with one exception: no interruption in RNA coverage was found at any point between *murB* and *ftsQ,* and no RNA initiation sites were consequently reported in this intergenic region [14].

We did instead find cDNAs terminating in this location in *B. mycoides.* Our experimental data, obtained by PE and RT-PCR, are thus in keeping with the results reported in the literature, since we found transcripts made up of five genes: *murG, murB, ftsQ, ftsA* and *ftsZ.* Moreover, the Northern blot showed *ftsZ* and *ftsA* RNA in the form of monogenic mRNAs, as well as of *ftsA-ftsZ*, *ftsQ-ftsA-ftsZ* and *murB-ftsQ-ftsA* RNAs.

### The *spoIIG* operon

The *B. mycoides dcw* cluster is closely followed by three genes expressed by the same DNA strand, forming a group homologous to the *spoIIG* operon that has been extensively characterized in *B. subtilis*[15-17]. The first gene, *spoIIGA*, encodes the protease required to activate the product of the second gene, *pro-sigmaE,* synthesized as an inactive precursor with an N-terminal prosequence. In *B. subtilis*, the region located between the *dcw* and the *spoIIG* clusters carries the high molecular weight *bpr* gene, a bacillopeptidase. In SIN and DX, the region between these clusters is short, non-coding and of different length (respectively 260 and 415 bp), and is identical along 70 nucleotides after *ftsZ* and 145 nucleotides before *SpoIIGA.* Only *B. weihenstephanensis*, in the *B. cereus* group, harbors a 415 bp spacer 100% identical to that of the DX strain, which points to the phylogenetic linkage of these two bacilli.

As the vicinity of the two clusters *dcw* and *spoIIG* might have a functional meaning, we searched for transcripts linking their genes. RT was performed with the BigD oligonucleotide (Table 1), which anneals at +273 relative to the first in frame ATG of the s*igmaE processing peptidase* (*SpoIIGA*). The primer was elongated up to −97 bp upstream of the *spoIIGA* ATG, in the spacer region that is identical in the *B. mycoides* DX and SIN strains. In the DX strain only, a higher band mapped inside the 3’ coding region of *ftsZ*. No elongation products included the complete *ftsZ* gene, thereby excluding a co-transcription of genes belonging to the two clusters (Additional file 2).

## Conclusions

Here we show that the organization and transcription of the *dcw* genes in the *B. mycoides* DX and SIN strains is not dissimilar, if we exclude minor variations that are most likely irrelevant to colony shape. Although only bicistronic transcripts were reported in *B. subtilis*, the novel finding is that *ftsZ* RNA is expressed as a single-gene transcript in the vegetative cells of these Gram positive bacilli. Multigenic *ftsZ* transcripts are also present, connecting the division genes to the upstream genes encoding enzymes of peptidoglycan biosynthesis. No common transcript was instead found between *ftsZ* and the downstream genes of the *SpoIIG* cluster.

## Methods

### Strains

*B. mycoides* DX and SIN are sporogenic bacilli of the soil isolated from the environment and maintained in the lab [3].

### RNA extraction

Exponentially growing cells were sedimented by centrifugation and blocked on ice in cell stop solution (5% phenol in ethanol). Cells were pelleted, suspended in 50% phenol in LETS buffer (10 mM Tris–HCl pH 8.0, 10 mM EDTA, 1% SDS, 10 mM DTT) and mechanically broken by vortexing with glass beads. After sedimentation at maximum speed in Eppendorf centrifuge, the supernatant was extracted twice with pH 4.3 phenol, then twice with chloroform and precipitated with 2M ammonium acetate final concentration and 1 volume isopropanol. The pellet was washed with 80% ethanol, air-dried and resuspended in RNAse-free water. DNA was digested with DNAseI (Amplification Grade, Invitrogen), according to the manufacturer’s indications.

### Analysis of RNA

Northern blots: electrophoresis of RNA in agarose-formaldehyde gels, blot and hybridization were conducted as described in Sambrook et al. [18]. The *ftsZ* specific DNA probe (551 bp) was obtained by PCR amplification of *B. mycoides* SIN DNA with primers Zfor and Zrev and the *ftsA* probe (408 bp) with primers Ain and N2R (Table 1). Amplified DNAs were labelled using a nick-translation kit (Boehringer).

For primer extension analysis, oligonucleotide primers were labeled at the 5’ end using T4 polynucleotide kinase and [γ-^32^P]ATP according to standard protocols [18]. 4 pmol of labeled oligonucleotides and 10 μg RNA were coprecipitated, suspended in 30 μl formamide buffer (10 mM PIPES pH 6.4, 0.1 M NaCl 0.1 mM EDTA 80% formamide) and incubated for 3 hrs at 30°C for annealing. Samples were diluted with 5 volumes water and precipitated with 0.25 M NaCl and ethanol. After 80% ethanol washing, the samples were dried in the air, suspended in 20 μl Super Script II Reverse Transcriptase buffer (Invitrogen) plus 10 mM DTT, 0.5 mM dNTP and 20 units RT and incubated at 42°C for 90 min. The enzyme was inactivated by heating at 70°C for 10 min and the RNA complementary to the cDNA digested away with RNAse H. Samples were precipitated with 0.25 M NaCl and ethanol, sedimented, washed with ethanol, dried and resuspended in 4 μl formamide-dye for electrophoresis on 6% acrylamide sequencing gels [18].

### RT-PCR

Two cDNAs were prepared using RNA purified from DNA (see above), one using the primer Zfin, which is complementary to the end of *ftsZ*, and a second using the primer *Afin*, which is complementary to the 3’ region of *ftsA*. The RNA was coprecipitated with the primers, suspended in formamide buffer, annealed and reverse transcribed as described above. The Zfin cDNA was amplified with Zfin as downstream primer and with Ain, Qin, Mbin, MGin and FW as upstream primers. The Afin cDNA was also amplified with Mbin and Qin. These primers are shown in Table 1. Control cDNA preparations were also prepared, omitting Reverse Transcriptase, to monitor possible residual DNA, and amplified. The PCR conditions were: 52°C for annealing and 7.5 min for elongation.

### Construction and expression of the *ftsZ* minigene

The DX region from coordinates 5947 to 6271 (GenBank AY129555), containing 246 bp of the 3’ *ftsA* coding region, the 39 bp *ftsA/ftsZ* spacer and the initial 39 bp of *ftsZ*, was amplified with primers which added an upstream *HindIII* site and a downstream *EcoRI* site (primers Hind5 and Eco5, see Table 1) and restricted at those sites. The DNA region containing the final 121 bp of the *ftsZ* ORF and 28 bp after the termination codon (coordinates 7267 to 7415) was amplified with the primers Eco3 and Bam3 (Table 1) that carry *EcoRI* and *BamHI* sites, respectively, and was restricted. Plasmid pJPR1 [9] (*‘amyE cat P*_*xyl*_*amyE’ bla,* a gift from J. Rawlings) was digested with *HindIII* and *BamHI* in the polylinker region, ligated to the prepared DNA fragments and transformed into *E. coli* Hb101. The correct recombinant plasmid was chosen by sequencing and used to transform competent *B. subtilis* 168. The *ftsZ* minigene became integrated at the *amyE* site as a result of a double crossing-over event between the 5’ and 3’ *amyE* regions carried upstream and downstream of the cloning site in pJPR1. Integration was controlled by sequencing. RNA transcribed from the minigene in the recombinant *B. subtilis* 168 was detected by primer extension with primer Amy5 (Table 1) annealing to the 5’ region of the *amyE* locus, 245 nucleotides downstream of the inserted minigene. Induction of the *pxyl* promoter by 5% xylose in TS was for 18 h and 3 h.

### Termination sequences

The putative *B. mycoides* termination sequences were detected on the basis of their identity to those predicted for *B. weihenstephanensis* at the TransTerm-HP site (*http://transterm.cbcb.umd.edu.).* The region of the *B. weihenstephanensis* KBAB4 genome considered was from coordinates 3780796 to 3790953 (Accession NC_010184), containing the genes of the *dcw* cluster from *murD* to *ftsZ* and the following *spoIIG* operon.

### Sequence data

Sequences of the *B. mycoides* SIN and DX partial *dcw* clusters are deposited as GenBank AY129554 (SIN) and AY129555 (DX).

## Competing interests

The authors declare that they have no competing interests.

## Authors’ contributions

TS participated in the design of the study and carried out the experiments. LT and GC added new data and confirmed previous data. CDF participated in the design and coordination of the study. EB conceived the study, organized the sequence data and drafted the manuscript. All the authors read and approved the final manuscript.

## Supplementary Material

Additional file 1 **Putative initiation sites of *ftsQ*, *ftsA *and *ftsZ *RNA as determined by primer extension.** The gene sequences are those of the *B. mycoides* DX strain (accession AY12555.2). The DNA complementary to the PE primers is highlighted in turquoise, as are the nucleotides of RNA start. Initiation and termination codons of the ORFs are in red. The hexamers corresponding to consensus TATA-box promoter motifs (17) and the ribosome binding sites are underlined. Click here for file

Additional file 2 **Determination of *SpoIIGA *RNA 5’ ends by Primer Extension.** The three genes of the *SpoIIG* cluster are encoded downstream of the *dcw* cluster, by the same DNA strand. The distance between the two clusters is 415 bp in DX and 260 bp in SIN. Primer extension started from primer BigD at position +273 of the first gene of the cluster, *SpoIIGA.* The DX and SIN cDNAs (two lanes each) were both elongated to position −97 upstream of the *SpoIIGA* first codon ATG, in the spacer region that is identical in both strains. A second cDNA termination, present only in DX, mapped within the 3’ end of the *ftsZ* coding region at −950. Click here for file
