# Supplementary figures and images for: Transcriptional analysis of ftsZ within the dcw cluster in Bacillus mycoides
Source: BMC Microbiol. 2013 Feb 6;13:27. doi: 10.1186/1471-2180-13-27 (PMC3762067; doi:10.1186/1471-2180-13-27)

## Slide 1
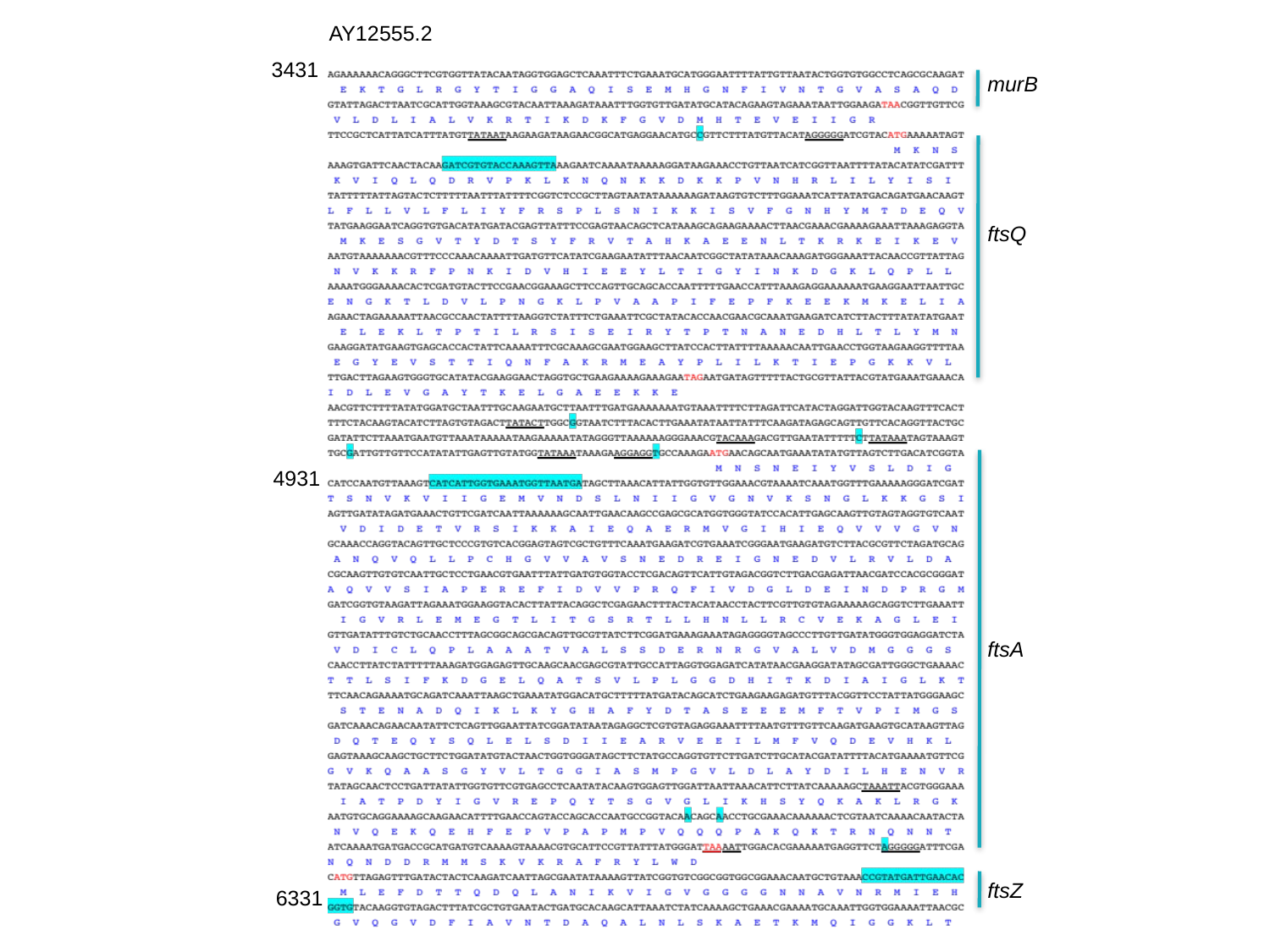

AY12555.2
3431
murB
ftsQ
4931
ftsA
ftsZ
6331

Supplement: Additional file 1 — Putative initiation sites of ftsQ, ftsA and ftsZ RNA as determined by primer extension. The gene sequences are those of the B. mycoides DX strain (accession AY12555.2). The DNA complementary to the PE primers is highlighted in turquoise, as are the nucleotides of RNA start. Initiation and termination codons of the ORFs are in red. The hexamers corresponding to consensus TATA-box promoter motifs (17) and the ribosome binding sites are underlined. [file 1471-2180-13-27-S1.pptx]

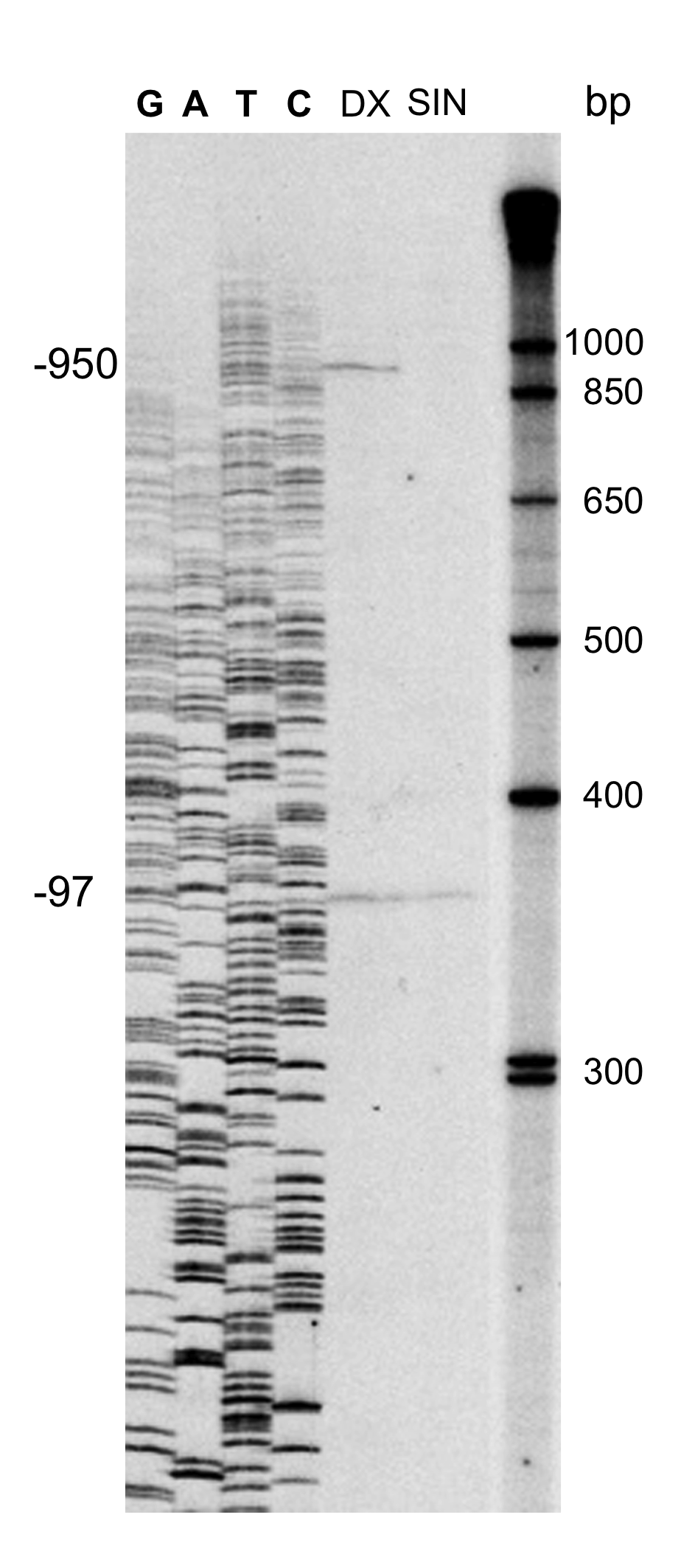

Supplement: Additional file 2 — Determination of SpoIIGA RNA 5’ ends by Primer Extension. The three genes of the SpoIIG cluster are encoded downstream of the dcw cluster, by the same DNA strand. The distance between the two clusters is 415 bp in DX and 260 bp in SIN. Primer extension started from primer BigD at position +273 of the first gene of the cluster, SpoIIGA. The DX and SIN cDNAs (two lanes each) were both elongated to position −97 upstream of the SpoIIGA first codon ATG, in the spacer region that is identical in both strains. A second cDNA termination, present only in DX, mapped within the 3’ end of the ftsZ coding region at −950. [file 1471-2180-13-27-S2.png]
